# Comparison of models for estimating methane emission factor for enteric fermentation of growing-finishing Hanwoo steers

**DOI:** 10.1186/s40064-016-2889-7

**Published:** 2016-07-29

**Authors:** Namchul Jo, Jongnam Kim, Seongwon Seo

**Affiliations:** Department of Animal Biosystem Sciences, Chungnam National University, 99 Daehak-ro, Yuseong-gu, Daejeon, 305-764 Republic of Korea

**Keywords:** Methane emission factor, Enteric fermentation, IPCC guidelines, Hanwoo (Korean native cattle)

## Abstract

The methodology provided by the Intergovernmental Panel on Climate Change (IPCC) guidelines is widely used for estimating enteric methane (CH_4_) production by cattle. No attempt other than the default values in the IPCC Tier 1 has been made for estimating CH_4_ emission from Hanwoo, a dominant beef species in Korea raised in a unique feeding system. The objective of this study was to compare models for estimating the CH_4_ emission factor (MEF; kg CH_4_/head/year) for enteric fermentation in Hanwoo steers. The MEF was estimated based on Korea- and Hanwoo-specific data obtained from the literature using several models. The models include the IPCC Tier 1 (T1), the IPCC Tier 2 method (T2), the IPCC Tier 2 methodology with actual dry matter intake (T2DMI), and the Japanese Tier 3 method (JT3). The JT3 was included due to the similarity in the beef cattle production system between the two countries. Estimated MEF using T2 were 43.4, 33.9, and 36.2 kg CH_4_/head/year for the growing, finishing, and overall period, respectively. The overall MEF estimated using T2 was 23 % lower than the estimate by T1 (47.0 kg CH_4_/head/year). There were significant differences in the estimated MEF for enteric fermentation of Hanwoo steers among the methods (*P* < 0.05). The overall MEF estimated by JT3 was 69.1 kg CH_4_/head/year, which was significantly higher than the estimates by T2 (36.2 kg) and T2DMI (33.5 kg). The JT3 estimated the highest values in all periods possibly due to overestimation of the conversion ratio of feed energy to CH_4_. No significant difference was found in the overall MEF of Hanwoo steers between T2 and T2DMI. However, T2DMI estimated 8 % higher and 14 % lower MEF than T2 for the growing and finishing period, respectively, mainly because the T2 significantly over-predicts the gross energy intake of Hanwoo steers at the high level of intake. The IPCC default methods have limitations in their use for a feeding systems in non-western countries, and thus development of a country-specific methodology and parameter estimates for enteric CH_4_ production is required for Hanwoo and other cattle production systems.

## Background

Due to an increase in public concern about climate change, greenhouse gas (GHG) emissions have become one of the major issues in all industrial sectors (Lashof and Ahuja 1990; Canadell et al. 2007; Meinshausen et al. 2009). Agricultural activity accounts for about 60 and 50 % of the global anthropogenic nitrous oxide (N_2_O) and methane (CH_4_) emissions, respectively, and the livestock sector has become recognized as an important contributor to GHG emissions (McMichael et al. 2007; Gerber et al. 2013). Enteric fermentation of cattle is the largest source of CH_4_ emissions in the livestock sector (Steinfeld et al. 2006). Accurate estimation of enteric CH_4_ production by cattle is thus required in order to develop a national GHG inventory and to establish mitigation strategies for GHG emissions from livestock production.

For the estimation of enteric CH_4_ production by cattle, methodologies suggested by the Intergovernmental Panel on Climate Change (IPCC) guidelines are widely used. The IPCC guidelines provide methodologies for estimating the enteric CH_4_ emissions from cattle at three levels of detail from Tier 1 (default values), Tier 2 (includes consideration of diet and energy intake), to Tier 3 (country specific methodology and parameter estimates). The Tier 1, the least precise approach, provides tabular fixed values. Although some countries (e.g., Germany, EU, Australia, Japan, the Netherlands) use a country-specific methodology/Tier 3 approach, the Tier 2 methodology is commonly used for quantifying the enteric CH_4_ emissions from cattle in many other countries for National Inventory Reports (NIR) (UNFCCC 2014). The IPCC Tier 2 approach estimates CH_4_ emissions from enteric fermentation of individual cattle by calculating a CH_4_ emission factor (MEF, kg CH_4_/head/year). This is the product of a CH_4_ conversion factor (MCF; percentage of gross energy [GE] in feed converted into CH_4_) and daily GE intake (MJ/head/day). The animal and feed characteristics are used to predict daily GE intake of cattle using equations, while pre-defined default values (0, 3.0, and 6.5 % for calves, feedlot, and the other stages of cattle, respectively) are used for MCF.

The Hanwoo is an indigenous and dominant cattle breed for beef production in South Korea. Hanwoo steers are raised for more than 28 months (normally weaned at 6 month old, growing phase for 6 months, and finishing pase for 16 months) for yielding a high quality beef with intense marbling. Hanwoo production has been recognized as a key source of GHG emissions from the agricultural sector in Korea; however, no attempt has been made for estimating CH_4_ emission from enteric fermentation of Hanwoo using methods other than the default values in the IPCC Tier 1 (GIR 2014). Furthermore, the equations provided by IPCC have been empirically developed on the basis of experimental data conducted mostly in western countries (e.g., USA and UK) (IPCC 2006). Since the feeding management of Hanwoo is much different (e.g., a much longer finishing period) from that of beef cattle in those countries, it may not be appropriate to use the IPCC equations for estimating enteric CH_4_ emissions for Hanwoo production.

The objectives of the current study was to compare models for estimating MEF for enteric fermentation of Hanwoo steers. Korean and Hanwoo specific data were obtained from the literature, and MEF for enteric fermentation of Hanwoo steers was estimated using several methods including IPCC Tier 1, IPCC Tier 2 methodology and Japanese Tier 3 method.

## Methods

### Estimation of MEF using the IPCC Tier 2 approach

Detailed description of the equations to estimate MEF using the IPCC Tier 2 approach is presented in the IPCC guidelines (2006). The IPCC Tier 2 approach estimates MEF using the following equation:

$${\text{MEF}} = ({\text{GEI}} \times ({\text{MCF}}/100) \times 365)/55.65,$$

where MEF is CH_4_ emission factor (kg CH_4_/head/year), GEI is daily gross energy intake (MJ/head/day) and MCF is CH_4_ conversion factor (%).

For the IPCC Tier 2 method (T2), daily GEI is calculated based on the net energy (NE) requirement of an animal and the digestible energy (DE) as a percentage of GE content of a diet (DE%) as described in the IPCC guidelines (IPCC 2006). The NE requirement of an animal is the sum of the requirements to support each physiological function (i.e., maintenance, activity, growth, lactation, pregnancy, work and wool production). Equations for estimating each NE requirement based on animal characteristics are provided in the IPCC guidelines (IPCC 2006). In addition, the IPCC guidelines suggest using the default constants for MCF: 0, 3.0, and 6.5 % for calves, feedlot and the other stages of cattle, respectively.

In addition, MEF was estimated using the IPCC Tier 2 approach with estimated GE intake based on actual DMI (T2DMI). For T2DMI, the same methodology in the IPCC Tier 2 was applied; however, GE intake was not calculated from NE requirement and DE%, but estimated based on actual DMI and estimated GE content of the diets. This estimated GE intake should be very close to the actual GE intake as GE contents are similar among normal diets (Maynard et al. 1979).

### Estimation of MEF using the Japanese Tier 3 method

The Japanese Tier 3 method (JT3) should be a reasonable method due to the similarities between Korea and Japan in terms of breeds, feed ingredients (mainly agricultural by-products), climate, and the duration of beef cattle on feeding to market weight (>28 months of age). The Japanese Tier 3 method estimates MEF using equations derived from country-specific experimental data (GIO 2014). It estimates daily enteric CH_4_ emissions of cattle on the basis of the predicted DMI using the following equations (GIO 2014):

$${\text{DMI}} = - 3.481 + 2.668 \times {\text{ADG}} + 4.548 \times 10^{ - 2} \times {\text{BW}} - 7.207 \times 10^{ - 5} \times {\text{BW}}^{2} + 3.867 \times 10 ^{ - 8} \times {\text{BW}}^{3}$$

$${\text{Y}} = - 17.766 + 42.793 \times {\text{DMI}}{-}0.849 \times {\text{DMI}}^{2}$$

$${\text{MEF}} = {\text{Y}}/22.4 \times 0.016 \times 365$$

where DMI is the daily dry matter intake (kg/day), ADG is the average daily gain (kg/day), BW is the animal live body weight (kg), Y is the daily enteric CH_4_ emission of a head of cattle (l CH_4_/head/day), and MEF is the CH_4_ emission factor (kg CH_4_/head/year).

### Comparison of MEF estimated by models for enteric fermentation of growing-finishing Hanwoo steers using models

The IPCC Tier 1 (T1), T2, T2DMI, and JT3 were used to estimate MEF for enteric fermentation of Hanwoo steers. The default MEF for the category of other cattle in North America (IPCC 1997) was used for T1. This is the value reported in the NIR of Korea (GIR 2014).

Using each of the T2, T2DMI, and JT3 methods, average GE intake (T2 and T2DMI), DMI (JT3), and eventually MEF for enteric fermentation of Hanwoo steers was estimated for each month throughout the typical feeding period for Hanwoo steers: the growing (6 months) and finishing (16 months) periods. Based on MEF estimated for each month, and the mean MEF was also calculated for the growing (6 months), finishing (16 months) and overall periods (22 months).

The animal and diet characteristics were obtained from Kim et al. (2005), which conducted a comprehensive feeding trial with 90 Hanwoo steers (three treatment means [n = 30] for 22 months). This is, to the best of our knowledge, the only study conducted in a typical commercial farm using a large number of the Hanwoo steers and measured body weight (BW) and dry matter intake (DMI) of the steers monthly throughout the feeding period. The average BW and average daily gain (ADG) was 261.6 kg and 766.8 g during the first 6 months of growing, and 519.3 kg and 845.9 g during the 16 months of finishing periods, respectively.

Dry matter intake and MEF of each month were estimated based on the reported BW and ADG for the JT3 method. In order to estimate DE% for both T2 and T2DMI, the DE and GE content (MJ/kg) of diets were calculated based on the reported nutrient composition. Digestible energy was converted from total digestible nutrient content (TDN, g/kg DM) of the diets multiplying by 0.00171 (NRC 2001). The GE content (MJ/kg) of the concentrate mixes was calculated based on the chemical composition:

$${\text{crude}}\;{\text{protein }}\left( {{\text{g}}/{\text{kg DM}}} \right) \times 2.34 + {\text{ether}}\;{\text{extract}}\left( {{\text{g}}/{\text{kg}}\;{\text{DM}}} \right) \times 3.93 + {\text{carbohydrate}}\left( {{\text{g}}/{\text{kg}}\;{\text{DM}}} \right) \times 1.76.$$

Carbohydrate content (g/kg DM) was calculated by:

$$1000{-}{\text{crude}}\;{\text{protein}}\left( {{\text{g}}/{\text{kg}}\;{\text{DM}}} \right){-}{\text{ether}}\;{\text{extract}}\left( {{\text{g}}/{\text{kg}}\;{\text{DM}}} \right) - {\text{ash}}\left( {{\text{g}}/{\text{kg}}\;{\text{DM}}} \right).$$

The nutrient composition of forages (i.e., rice straw and orchard grass) was unknown (Kim et al. 2005); therefore, the GE value of the forages was calculated on the basis of the mean chemical composition of forages obtained from the Korea standard feed composition table (NIAS 2012b).

Based on this information, NE requirements of the steers, NE available in diet for growth and maintenance, DE as a percentage of feed GE, and eventually GE intake was calculated using the equations in the IPCC guidelines (Table 1). The default MCF values (6.5 and 3.0 % for growing and finishing period, respectively) were used in the MEF estimation for both T2 and T2DMI.

**Table 1:** Descriptive statistics of the data used to estimate gross energy intake of growing-finishing Hanwoo steers based on the IPCC Tier 2 method

	Mean	SD	Coefficient of variation (%)
Body weight (kg)			
Growing period	261.6	35.3	13.5
Finishing period	519.3	118.7	22.9
Average daily gain (g/day)			
Growing period	766.8	163.1	21.3
Finishing period	845.9	112.9	13.4
Net energy requirement for maintenance (NEm, MJ/day)			
Growing period	20.2	2.2	10.9
Finishing period	34.2	6.1	17.8
Net energy requirement for growth (NEg, MJ/day)			
Growing period	10.1	2.4	23.4
Finishing period	19.2	4.8	25.0
Ratio of net energy available for maintenance (REM)			
Growing period	0.524	0.001	0.163
Finishing period	0.530	0.004	0.813
Ratio of net energy available for growth (REG)			
Growing period	0.325	0.001	0.421
Finishing period	0.334	0.007	2.081
Digestible energy content (as a percentage of growth energy; DE%)^a^			
Growing period	68.4	0.3	0.4
Finishing period	70.5	1.7	2.3

The basal information of monthly animal body weight, average daily gain and diet information were obtained from Kim et al. (2005)

^a^Digestible energy as a percentage of gross energy content in a diet

For each period (i.e., growing, finishing, and overall periods), the MEF estimated from all three methods (i.e., T2, T2DMI, and JT3) were compared with PROC MIXED (SAS, Institute, Cary, NC, USA), with each month as a block. Pair-wise comparisons of the least square means was conducted using the PDIFF option with a Tukey–Kramer adjustment when a significant (*P* < 0.05) difference among three methods was observed. The linear model was as follows:

$$y_{ijk} = {{\upmu }} + \alpha_{i} + b_{j} + e_{ijk} ,$$

where y_ijk_ is the estimated CH_4_ emission factor, μ is the overall mean, α_i_ is the fixed effect of the *i*th method, b_j_ is the random effect of the *j*th month, and *e*_ijk_ is the unexplained random error.

### Evaluation of intake models

Since the most important variable for estimating MEF is GE intake in the IPCC Tier 2 approach and DMI in the Japanese Tier 3 method, the two intake models were evaluated using the actual DMI of Hanwoo steers. The GE intakes predicted by the IPCC Tier 2 method were compared with those estimated based on the actual DMI to evaluate the predictability of the GE intake prediction model in the IPCC Tier 2. In addition, the Japanese Tier 3 model for predicting DMI was also evaluated using the actual DMI of Hanwoo steers. In both evaluations, observed values were regressed against predicted values. For the evaluation of the GE intake model in the IPCC Tier 2, the GE intakes estimated from the actual DMI were assumed as observed values. The coefficient of determination (*R*^2^) was used to assess the precision of the intake models. The root mean square prediction error (RMSPE; Bibby and Toutenburg 1977), was used to determine the accuracy of the intake models. Residual analyses were also conducted to assess the slope and mean biases of the prediction, as proposed by St-Pierre (2003). The predicted values were centered around the mean predicted values before the residuals were regressed on the predicted values.

## Results

The reported enteric MEF of Hanwoo (T1) was 47.0 kg CH_4_/head/year (GIR 2014), the default value for the category of other cattle in North America (IPCC 1997). Based on the animal and diet information from a comprehensive study by Kim et al. (2005), the enteric MEF of Hanwoo steers estimated separately for the growing, finishing, and overall feeding periods using the IPCC Tier 2 method (T2) were 43.4, 33.9, and 36.2 kg CH_4_/head/year, respectively (Table 2). The overall MEF estimated by T2 was 23 % lower than T1.

**Table 2:** Estimated enteric methane emissions factor (MEF, kg CH_4_/head/year) using different methods for growing-finishing Hanwoo steers

	Methods for estimating MEF^a^			SEM	*P* value
	T2	T2DMI	JT3		
Growing	43.4^d^	46.8^c^	57.1^b^	0.90	<0.001
Finishing	33.9^c^	29.3^d^	72.8^b^	0.50	<0.001
Overall	36.2^c^	33.5^c^	69.1^b^	1.39	<0.001

^a^T2; the IPCC Tier 2 method (IPCC 2006), T2DMI; the IPCC Tier 2 methodology using gross energy intake estimated from dry matter intake instead of using the gross energy intake predicted by the IPCC Tier 2 model, JT3; The Japanese Tier 3 method (GIO 2014)

^b,c,d^Means that do not have common superscripts differ (*P* < 0.05)

There were significant differences in the estimated MEF for enteric fermentation of Hanwoo steers among the T2, T2DMI, and JT3 methods (*P* < 0.05, Table 2). The values estimated using JT3 were the highest in all cases (i.e., growth, finishing, and overall). The largest discrepancy was observed in the finishing period; the estimated enteric MEF for finishing using JT3 was 115 and 148 % higher than that using T2 and T2DMI, respectively. Between T2 and T2DMI, we found no significant difference in the overall MEF of Hanwoo steers. However, there were significant differences in MEF for both growing and finishing periods between the two methods. Compared to T2, the T2DMI was 8 % higher and 14 % lower for estimating MEF for the growing and finishing period, respectively.

**Fig. 1:**
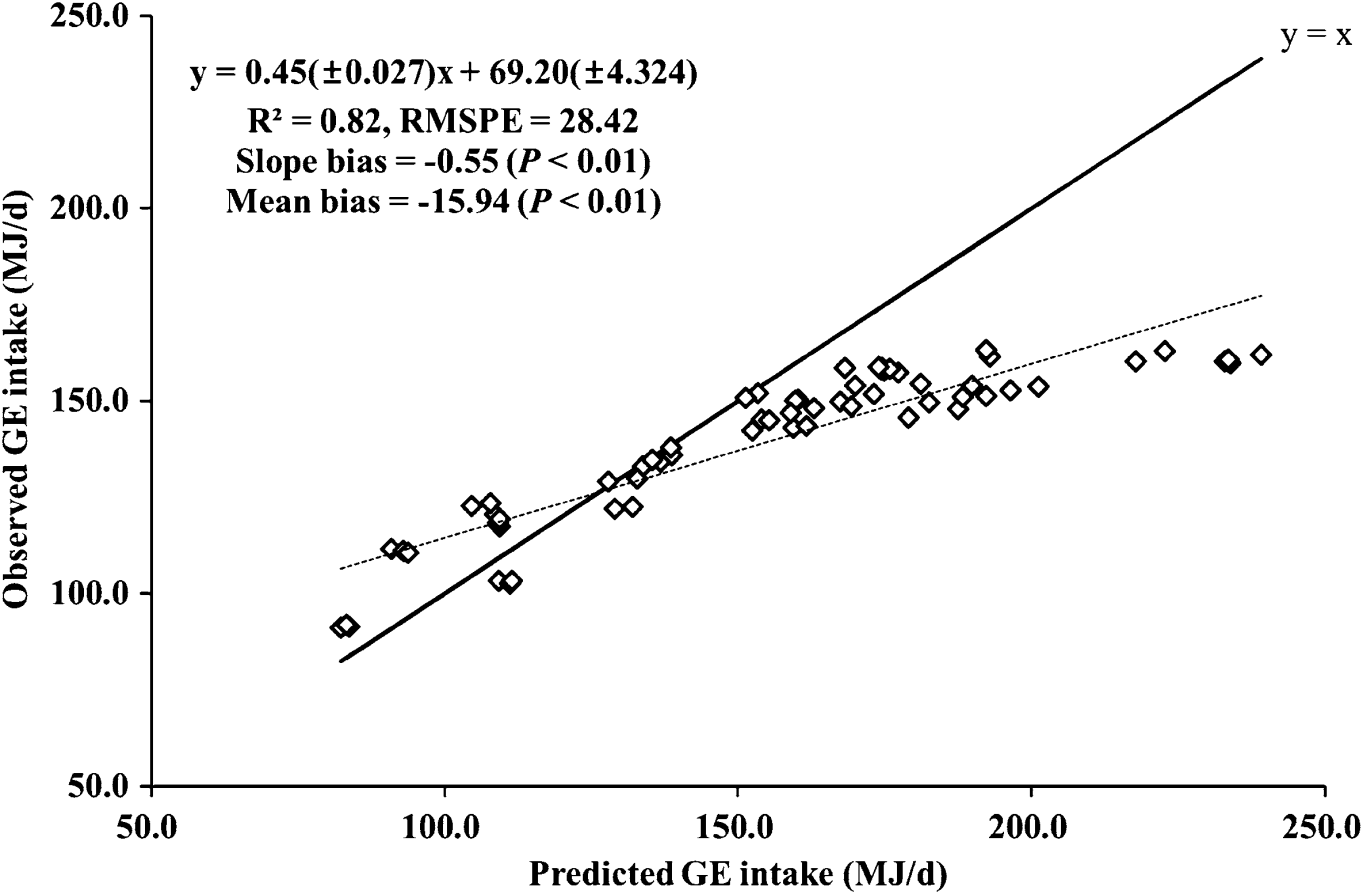
Regression of observed and predicted gross energy (GE) intake (MJ/day) using the IPCC Tier 2 model. The GE intake estimates were based on the actual DMI and were assumed to be observed values. The *solid* and *dotted lines* represent y = x and the best-fit linear regression, respectively, and the regression equation (*dotted line*) is presented. RMSPE is root mean square prediction error

The differences between T2 and T2DMI are likely to be due to the differences in GE intake since the same MCF were used in both methods. The mean bias of the GE intake prediction model in the IPCC Tier 2 was statistically significant at 10 % (Fig. 1). There was also a significant bias in slope, resulting in the IPCC Tier 2 model underestimating GE intake when the level of intake was low (i.e., growing period), and overestimating it when the level of intake was high (i.e., finishing period). Moreover, the relationship between the observed and predicted values was curvelinear (Fig. 1), implying that the IPCC Tier 2 model overestimated GE intake as the level of intake increased. These biases were reflected in the estimation of MEF by T2DMI compared to T2; higher estimates during growing while much lower estimates during finishing. The overestimation of GE intake by the IPCC Tier 2 model at the high level of intake during the later stage of feeding is likely because the model was developed based on data from the US and UK, where most beef cattle are raised for a shorter period of time than in Korea. This result implies that the GE intake model provided in the IPCC guidelines may not be appropriate to be used for Hanwoo steers due to the uniqueness of the Hanwoo production system.

**Fig. 2:**
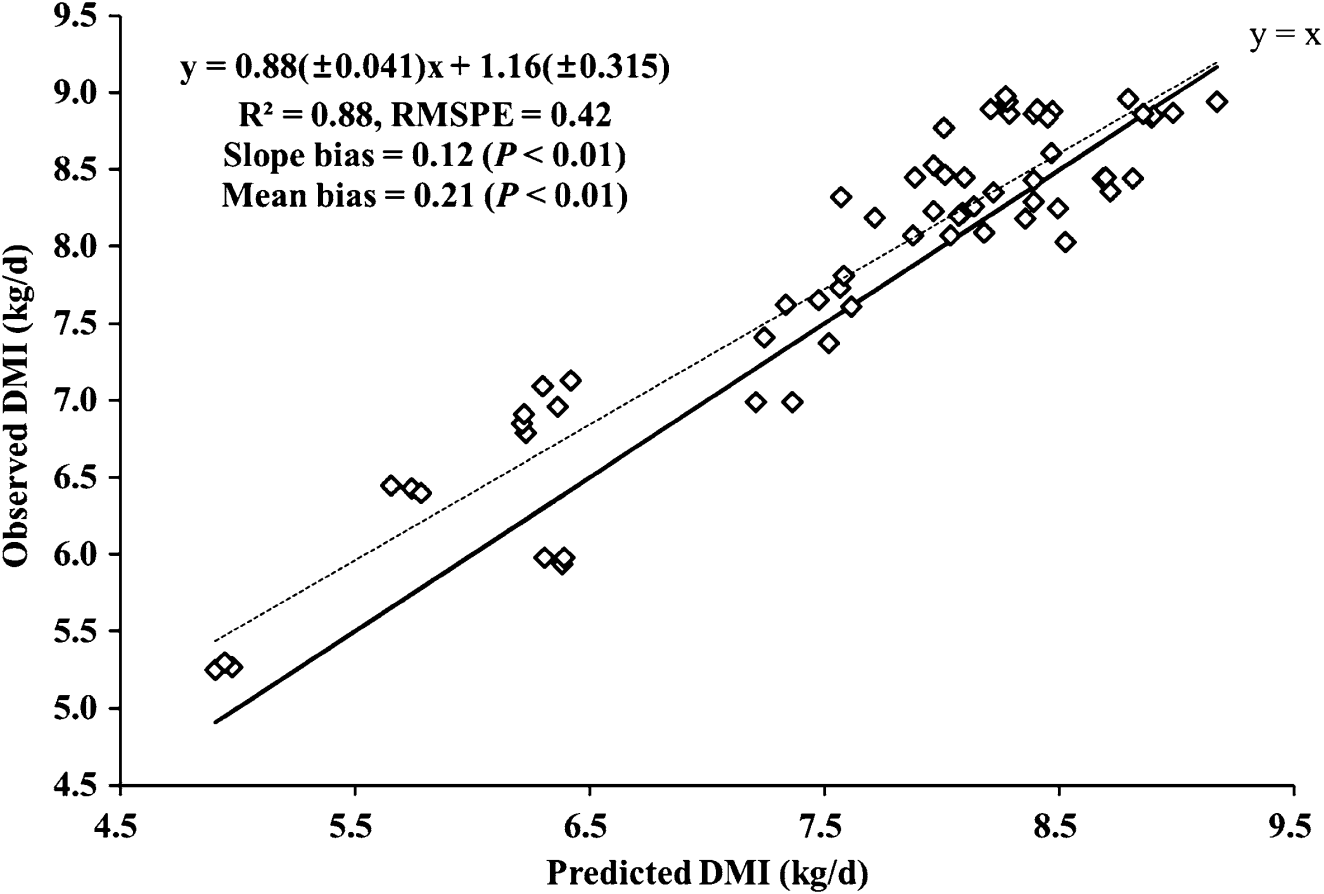
Regression of observed and predicted dry matter intake (DMI, kg/day) using the equations presented in the Japanese Tier 3 method. *Solid* and *dotted lines* represent y = x and the best-fit linear regression, respectively, and the regression equation (*dotted line*) is presented. *RMSPE* root mean square prediction error

The DMI equation in the Japanese Tier 3 method predicted DMI of Hanwoo steers surprisingly well, although the estimated MEF by JT3 were much higher than those by T2 and T2DMI (Fig. 2). Although the DMI model was derived from the experimental data on Japanese cattle, it explained 88 % of the variations in the observed DMI of Hanwoo steers (RMSPE of 0.42). The coefficient of variation of the predictions was only 5.5 %. This supported the possibility of applying the DMI prediction model for Japanese beef cattle for predicting DMI of Hanwoo steers due to the similarity in terms of the origin of the breeds, feed ingredients, climate, and the duration of feeding before harvest.

## Discussion

Quantification of CH_4_ emissions from enteric fermentation of cattle is required for filing national GHG inventory reports and searching for possible mitigation strategies to reduce GHG emissions from cattle production. For this purpose, the IPCC has developed guidelines and methodologies to estimate GHG emissions from livestock ranging from Tier 1–3, based on the availability of country-specific data and models (IPCC 1997, 2006). Although the IPCC recommends use of the Tier 2 or 3 method, these methods require a more detailed characterization of the animals, diets, and management systems (IPCC 2006). This information may not be readily available in many countries, particularly where different production systems are applied compared to western countries (such as Korea). The default value in the IPCC Tier 1 was used when estimating CH_4_ emissions from Hanwoo production for the NIR of Korea (GIR 2014). The default value for the category of other cattle in North America (IPCC 1997) was used mainly due to similar productivity. The production of Hanwoo is a major source of GHG emissions from the agricultural sector in Korea (GIR 2014), and thus it is important to estimate CH_4_ emissions more accurately for reducing national GHG emissions and increasing the sustainability of Hanwoo production.

The IPCC Tier 1 relies on a fixed MEF crudely determined by regional characteristics and production levels. In contrast, the IPCC Tier 2 methodology predicts MEF on the basis of GE intake and MCF using a more mechanistic approach (IPCC 2006). Using the IPCC Tier 2 methodology, the enteric MEF of Hanwoo steers could be estimated separately for the growing, finishing, and overall feeding periods. This reduces uncertainty and is one of the important advantages of the Tier 2 over the Tier 1. The uncertainty in estimating MEF for enteric fermentation may determine that in estimating the CH_4_ emissions from the livestock production sector (Milne et al. 2014). The mechanistic approach used in the IPCC Tier 2 methodology allows the enteric CH_4_ production of cattle to be estimated while reducing uncertainty involving the animals, diets, and management characteristics (Ominski et al. 2007).

The MEF estimated by T2 was significantly smaller than T1. The large difference in the estimated MEF between T1 and T2 may be because Hanwoo stay in feedlots for a long period of time (>16 months). The MCF for the feedlot cattle is assumed to be 3 % of GE intake, which is much smaller than 6.5 % during normal feeding (IPCC 2006). T1 assumes the typical feeding situations in North America, and thus does not account for the reduction in enteric CH_4_ production during an extended finishing period in Hanwoo production. The MEF estimated by the IPCC Tier 2 is not always lower than the value estimated by Tier 1. Previous comparisons between the two methodologies in Canada indicated that the Tier 2 methodology was 25 and 19 % higher than the default values of the IPCC Tier 1 for beef bulls and steers >1 year, respectively (Basarab et al. 2005; Ominski et al. 2007). These results in addition to our study suggests that the Tier 2 methodology may be more appropriate than Tier 1 in terms of reflecting differences in a country specific feeding system.

Although the Tier 2 methodology is more appropriate than the Tier 1 approach (Höglund-Isaksson 2012), development of models and coefficients for a specific feeding system is required. For the overall feeding period of Hanwoo steers, the estimated MEF using T2 was similar to results using T2DMI, implying that the IPCC Tier 2 method may be applied for estimating enteric CH_4_ emissions from Hanwoo in filing NIR. However, there were significant differences in estimating the MEF separately for growing and finishing periods between T2 and T2DMI, indicating that the uncertainty in estimating MEF for enteric fermentation still remains in T2 (Bannink et al. 2011; Milne et al. 2014). The uncertainty in the MEF estimates for the IPCC Tier 2 methodology results from GE intake prediction and MCF. There have been several efforts to investigate the adequacy of the MCF values suggested by the IPCC guidelines and to revise them to be more accurate and representative of a specific diet condition (Kebreab et al. 2008; Ellis et al. 2010; Bannink et al. 2011). Furthermore, a reduction in MCF means an increase in efficiency for converting feed energy to metabolizable energy, and thus it has been of particular interest in recent cattle nutrition studies (McGinn et al. 2004; Beauchemin et al. 2007). Relatively little attention, however, has been directed to the IPCC equations for estimating GE intake. The equations provided by IPCC were empirically developed on the basis of the experimental data conducted mostly in western countries (e.g. USA and UK) (IPCC 2006). The model may thus not predict GE intake accurately in other feeding systems, as shown in the current study. Differences in breed and feeding management of Hanwoo resulted in biases in the predictions of GE intake by the IPCC Tier 2 model, particularly at a high level of intake.

In order to accommodate country-specific differences and to develop an appropriate mitigation strategy, some countries (e.g., Germany, EU, Australia, Japan, the Netherlands) have attempted to develop a country-specific methodology (the Tier 3 approach). Some of these country-specific models have incorporated dynamics of rumen digestion and various aspects of dietary characteristics on CH_4_ production (Benchaar et al. 2001; Bannink et al. 2011). Considering the uniqueness of the Hanwoo production system, development of a country-specific methodology and parameter estimates is required.

Since the beef cattle production system in Japan is similar to that in Korea, it was hypothesized that the Japanese Tier 3 method could be used for estimating enteric fermentation of Hanwoo steers. Even so, the accuracy and precision of the DMI model for Japanese cattle in predicting DMI of Hanwoo was higher than our expectation. The DMI model for Japanese cattle predicts intake of an animal using only BW and ADG, and was empirically developed on the basis of the data obtained from locally conducted experiments (GIO 2014). In many cases, an empirical model works specifically within the range of data on which the model was based, and a mechanistic approach is preferred when a predicted system is different from the system where the model was developed (Seo 2012). Since, to the best of our knowledge, there has been no study that has evaluated the DMI model for Japanese cattle in predicting the DMI of Hanwoo steers, the single experiment evaluation in this study may not be sufficient for drawing conclusions. Nevertheless, these results showed the potential for applying the DMI model of Japanese cattle for predicting the DMI of Hanwoo steers.

The JT3 method for estimating the MEF of Japanese cattle may overestimate that of Hanwoo steers. Since the DMI model for Japanese cattle predicted the DMI of Hanwoo steers relatively well, it was inferred that the main differences in the estimated MEF between JT3 and the other methods might be the over-prediction of JT3 in converting intake energy to CH_4_, MCF. To confirm this, the MCF was back-calculated from the MEF estimated using JT3. Based on JT3, the average MCF for growing and finishing was 7.9 and 7.5 % (ranged from 7.26 to 8.01 %), respectively. These were much higher than the default values in the IPCC Tier 2 and the values previously measured in Hanwoo steers. A study measuring CH_4_ emissions of growing Hanwoo steers using a hood-type chamber system, reported that MCF of growing Hanwoo steers was 5.5 and 6.5 % with corn- and barley-based diets, respectively (Seol et al. 2011). The same group also showed that the MCF of Hanwoo steers in feedlots was 5 and 4 % with corn- and barley-based diets, respectively (Seol et al. 2012). Since the average intake of the Hanwoo steers in these studies were lower than those in our study and field observations, the actual MCF of Hanwoo steers may be lower. These results imply that the MCF of Hanwoo steers may be less than what is estimated using JT3. Therefore, it is suggested that JT3 be inappropriate for estimating MEF of Hanwoo steers even though the DMI prediction model can be used to predict DMI of Hanwoo steers.

One of the limitations in this study is that the animal and diet characteristics were obtained from a single comprehensive study (Kim et al. 2005). The MEF values estimated in the study may thus not represent the national average in Korea. Nonetheless, the values were likely similar to those in the field since the diet and the growth rate of the steers used in this study are similar to those reported and suggested in the Korean Feeding Standard of Hanwoo (NIAS 2012a). Another limitation was that enteric CH_4_ production of Hanwoo steers was not actually measured. We intend to measure enteric CH_4_ in future studies when validating the results observed in the present study.

## Conclusions

The IPCC default methods have limitations in their use for a feeding systems in non-western countries possibly because the equations provided by IPCC have been empirically developed on the basis of experimental data conducted mostly in western countries. In order to reduce the uncertainty of the estimates and search for a better mitigation strategy, development of a country-specific methodology and parameter estimates for enteric CH_4_ production of Hanwoo is required.

## Abbreviations

BW: body weight
CH_4_: methane
DE%: digestible energy as a percentage of gross energy
DMI: dry matter intake
GE: gross energy
GEI: gross energy intake
GHG: greenhouse gas
IPCC: Intergovernmental Panel on Climate Change
JT3: the Japanese Tier 3 method
MCF: methane conversion factor
MEF: methane emission factor
NE: net energy
NIR: National Inventory Report
T1: the IPCC Tier 1 method
T2: the IPCC Tier 2 method
T2DMI: the IPCC Tier 2 methodology with actual dry matter intake
